# Efficacy and safety of rivaroxaban for the treatment of PICC-related upper extremity deep vein thrombosis in cancer patients: a retrospective study

**DOI:** 10.1186/s12959-023-00456-9

**Published:** 2023-02-01

**Authors:** Jiaxuan Xu, Guodong Wang, Xiaojie Chen, Yanfen Shen, Xinpeng Wang, Hongzhi Wang

**Affiliations:** Department of Critical Care Medicine, Key Laboratory of Carcinogenesis and Translational Research (Ministry of Education/Beijing), Peking University Cancer Hospital & Institute, Beijing, China

**Keywords:** Upper extremity deep vein thrombosis, Neoplasm, Anticoagulant therapy, Direct oral anticoagulants, Catheter-related thrombosis

## Abstract

**Background:**

The optimal duration and choice of anticoagulant for the treatment of Peripherally inserted central catheters (PICC)-related upper extremity deep vein thrombosis (UEDVT) in cancer patients are still undetermined.

**Objectives:**

The aim of this study was to assess the efficacy and safety of rivaroxaban for the treatment of PICC-related UEDVT in cancer patients.

**Methods:**

We conducted a retrospective cohort study including consecutive cancer patients for the management of acute symptomatic PICC-related UEDVT. The efficacy outcome of the study was the 180-day recurrence of any venous thromboembolism (VTE), while the safety outcome was the 180-day incidence of all bleeding events. The Kaplan‒Meier method was used to estimate the overall incidence. Hazard ratios (HRs) were obtained with a Cox proportional hazards model to estimate the risk of the outcome events.

**Results:**

A total of 217 patients were included in the final analysis with a median age of 56 years old, 41.5% of whom had metastases. After the initial 3–5 days of nadroparin, patients received sequential anticoagulation, either with nadroparin (118 patients) or with rivaroxaban (99 patients). Four patients with recurrent VTE were observed (nadroparin, *n* = 2; rivaroxaban, *n* = 2). The 180-day cumulative VTE recurrence rates were 1.7% and 2.0% (*p* = 0.777) in patients receiving nadroparin and rivaroxaban, respectively. The overall bleeding rate at 180 days was 8.8%. Although no major bleeding events were observed, nineteen patients with clinically relevant nonmajor bleeding (CRNMB) were observed. The 180-day cumulative rate of CRNMB was 5.1% for nadroparin and 13.1% for rivaroxaban (HR = 3.303, 95% CI 1.149–9.497, *p* = 0.027).

**Conclusion:**

Our study supported the efficacy of rivaroxaban for treating PICC-related UEDVT in cancer patients. However, data on anticoagulation therapy for PICC-related UEDVT presented with a low risk of VTE recurrence and a relatively high risk of CRNMB bleeding events. Considering the risk–benefit ratio, further well-designed trials are required to optimize the drug selection and duration for the treatment of PICC-related UEDVT in cancer patients.

## Introduction

Peripherally inserted central catheters (PICCs) are widely used in cancer patients because they are essential to the care of cancer-associated treatment, facilitating the delivery of cancer chemotherapy, stem cell reinfusion, parenteral nutrition, antibiotics and blood products. However, given the underlying hypercoagulable status, cancer patients are particularly vulnerable to catheter-related thrombosis. Catheter-related UEDVT is the most common noninfectious complication after PICC insertion, representing approximately 5%-10% of all cases of deep vein thrombosis (DVT) [1]. PICC-related UEDVT can cause consequences if not managed promptly, including catheter dysfunction, chronic venous occlusion, post-thrombotic syndrome, anti-cancer therapy interruption and rarely, pulmonary embolism (PE). Management of PICC-related UEDVT in cancer patients is more challenging than in the general population because cancer patients are both at higher risk of thrombosis recurrence and anticoagulation-associated bleeding events. In the past decade, direct oral anticoagulants (DOACs) have emerged as a new therapeutic option. Several multicenter randomized controlled studies comparing direct Xa inhibitors with LMWH for the treatment of cancer-associated venous thromboembolism (VTE) have been published to support the efficacy and safety of DOACs in clinical practice [2–5]. To date, randomized controlled trials for the treatment of PICC-related UEDVT in cancer patients are scarce, and current recommendations for the management are mostly extrapolated from limited observational cohorts and available data of noncancer patients or lower extremity DVT. For cancer patients with PICC-related UEDVT, major guidelines recommend low molecular weight heparin (LMWH) as first-line anticoagulation treatment for at least three months [6–8]. The optimal duration and choice of anticoagulation for catheter-related UEDVT in cancer patients remain unclear. The aim of this retrospective cohort study was to assess the efficacy and safety of rivaroxaban in the anticoagulation therapy of PICC-related UEDVT in cancer patients.

## Methods

### Patients and study design

We conducted a retrospective cohort study at Peking University Cancer Hospital, a tertiary care academic hospital in Beijing, China. Consecutive cancer patients diagnosed with acute symptomatic catheter-related UEDVT in our Venous Access Center between January 1, 2016, and December 31, 2020, were included. As a department of Peking University Cancer Hospital, the Venous Access Center was established in 2010 and staffed by a full-time team of doctors and nurses. The center provides services of the central venous access device placement (including centrally inserted central catheters, PICCs and totally implantable ports), regular maintenance and comorbidity management for both inpatients and outpatients. A specialized electronic data platform integrated with the electronic medical record system (EMR) was developed for catheter administration in 2016 to document details for every patient from catheterization to weekly maintenance until catheter withdrawal. All patient data were retrieved from this registry system.

Patients were included if they met all the following criteria: 1) at least 18 years of age; 2) active malignancy under anticancer therapy; and 3) newly diagnosed symptomatic PICC-related UEDVT. The diagnosis of PICC-related UEDVT required the evidence of a filling defect on compression ultrasonography, venography, CT or MR venography involving the ipsilateral internal jugular, brachial, axillary, subclavian, brachiocephalic or superior vena cava. We excluded patients with inherited or acquired thrombophilia and those with on-going anticoagulation. Patients started the treatment with a therapeutic weight-based dose (950 anti-Xa units/10 kg subcutaneously every 12 h) of nadroparin calcium immediately after the UEDVT diagnosis was confirmed. After the initial 3 to 5 days of therapy with nadroparin, anticoagulant can be transitioned to rivaroxaban (15 mg twice daily up to a total of 21 days anticoagulation, then change to maintenance dose of 20 mg once daily). According to the institutional protocol for the management of VTE, a standard 90-day anticoagulation with a therapeutic dose was recommended as long as the catheter was in place. Prolonged anticoagulation after 90 days was based on the treatment response, patient tolerance and discretion of the attending physician at Venous Access Center. Patients were scheduled with a follow-up of 180 days, with weekly clinical assessment and monthly ultrasonographic evaluation. An emergency imaging examination will be arranged in case of suspicion for recurrent VTE events.

### Study outcome

The efficacy outcome of this study was recurrent VTE at 180 days after the initiation of anticoagulation. Recurrent VTE was defined as any site of DVT or PE, with objective evidence of a new filling defect on compression ultrasonography, venography, or computed tomographic pulmonary angiography (CTPA). The safety outcome was a composite bleeding event of major bleeding (MB) and clinically relevant nonmajor bleeding (CRNMB) as defined by the International Society of Thrombosis and Hemostasis (ISTH) [9]. According to the definition established by ISTH, major bleeding events were defined as critical site (such as intracranial, intraspinal, intraocular, retroperitoneal, intra-articular or pericardial) bleeding, fatal bleeding, or overt bleeding with a fall of at least 20 g per liter in the hemoglobin level, resulting in the need for transfusion of 2 or more units of red blood cells. CRNMB events were defined as any sign or symptom of hemorrhage that did not fit the criteria for the ISTH definition of major bleeding but did meet at least one of the following criteria: 1) requiring medical intervention by a healthcare professional; 2) leading to hospitalization or increased level of care; 3) prompting a face-to-face evaluation.

### Statistics

Statistical analyses were carried out with SPSS software package, version 26 (IBM, NY, USA) and R software (version 4.2.1). Continuous variables with a normal distribution are described as the means with standard deviations, while continuous variables with a skewed distribution are presented as median values with interquartile ranges. Comparisons between two groups were performed with 2-tailed Student’s *t* test. Discrete variables are presented as frequencies and percentages, and group comparisons were performed using the chi-square or Fisher’s exact test. A *p* value < 0.05 was considered to indicate statistical significance. Cumulative incidence curves were estimated using Kaplan‒Meier curves and log-rank tests. Univariate analysis was used to screen baseline group differences in demographics and comorbidities. Factors with statistically significant differences were included for further multivariate Cox proportional hazards analysis. Proportional hazards assumptions were assessed by evaluation of Schoenfeld residuals for included variables. Hazard ratios (HRs) with associated 95% CIs were obtained to estimate the risk of the outcome events.

## Results

### Baseline characteristics

**Fig. 1:**
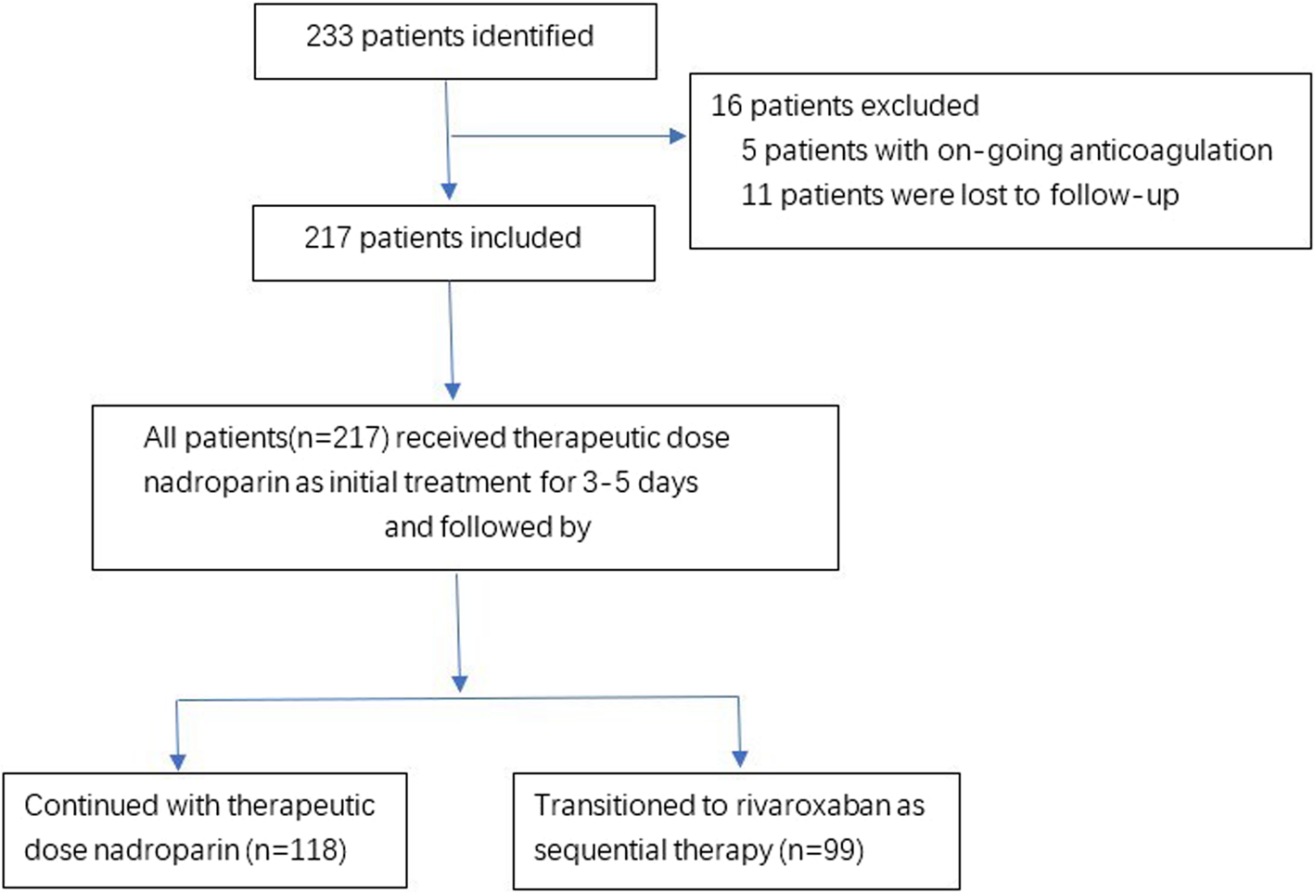
Flow diagram of the study

A total of 233 consecutive patients with confirmed catheter-related UEDVT were identified. Among them, 16 (6.9%) patients were excluded: 5 patients with ongoing anticoagulation and 11 patients who were lost to follow-up. Thus, 217 (93.1%) patients were included in the final analyses (Fig. 1).

The demographic and clinical characteristics of the study population are depicted in Table 1. The median age of the study population was 56 (48–63) years old, and 42.4% of patients were male. Ninety (41.5%) patients were documented with metastatic status, and 53 (24.4%) patients received antiangiogenic therapy for cancer treatment. Overall, patients were followed-up for a median of 185 days (range from 58 to 473 days).

**Table 1:** Patient demographics and comorbidities

	**Total (** ***n*** ** = 217)**	**Sequential treatment with nadroparin (** ***n*** ** = 118)**	**Sequential treatment with rivaroxaban (** ***n*** ** = 99)**	***p*** ** value**
**Age, median (IQR)**	56(48–63)	57(49–63)	56(46–62)	0.093
**Male gender (%)**	92(42.4%)	62(52.5%)	30(30.3%)	0.001
**BMI, median (IQR)**	23.0(21.0–26.4)	23.1(21.6–26.1)	22.9(20.5–27.4)	0.376
**With other site VTE**				
**Pulmonary thromboembolism**	8(3.7%)	4(3.4%)	4(4.0%)	1.000
**Lower extremity Deep vein thrombosis**	17(7.8%)	9(7.6%)	8(8.1%)	1.000
**VTE history**	13(6.0%)	8(6.8%)	5(5.1%)	0.776
**Bleeding history**	11(5.1%)	4(3.4%)	7(7.1%)	0.234
**Primary cancer**				< 0.001
** Breast**	59(27.2%)	25(21.2%)	34(34.3%)	
** Lymphoma**	40(18.4%)	33(28.0%)	7(7.1%)	
** Gastrointestinal**	61(28.1%)	42(35.6%)	19(19.2%)	
** Gynecology**	25(11.5%)	10(8.5%)	15(15.2%)	
** Lung**	32(14.7%)	8(6.8%)	24(24.2%)	
**With metastasis (%)**	90(41.5%)	45(38.1%)	45(45.5%)	0.276
**ECOG performance status**				0.002
** 0**	60(27.6%)	28(23.7%)	32(32.3%)	
** 1**	92(42.4%)	43(36.4%)	49(49.5%)	
** 2**	65(30.0%)	47(39.8%)	18(18.2%)	
**Antiangiogenic** **therapy used**	53(24.4%)	37(31.4%)	16(16.2%)	0.009
**Underlying diseases/conditions**				
** Cardiovascular disease**	41(18.9%)	20(16.9%)	21(21.2%)	0.424
** Diabetes**	22(10.1%)	18(15.3%)	4(4.0%)	0.007
** Abnormal renal function (30 ml/min ≤ CrCl < 60 ml/min)**	12(5.5%)	5(4.2%)	7(7.1%)	0.363
** Reduced platelet (Platelet count < 100 × 10^9/L)**	13(6.0%)	8(6.8%)	5(5.1%)	0.593
** Anemia(Hemoglobin < 130 g/L for man, < 120 g/L for woman)**	35(16.1%)	17(14.4%)	18(18.2%)	0.451
**Non-single lumen PICC**	8(3.7%)	6(5.1%)	2(2.0%)	0.295
**Left arm insertion**	34(15.7%)	26(22.0%)	8(8.1%)	0.005
**Suspect exit site infection**	9(4.1%)	3(2.5%)	6(6.1%)	0.306
**Treatment duration (days, IQR)**	180(125, 180)	180(120, 180)	180(126, 181)	0.229
**Outcome**				
**Recurrent VTE**	4(1.8%)	2(1.7%)	2(2.0%)	1.000
**Major bleeding**	0	0	0	NA
**Clinical related nonmajor bleeding**	19(8.8%)	6(5.1%)	13(13.1%)	0.037
**Death from any cause**	3(1.4%)	2(1.7%)	1(1.0%)	1.000

*BMI* body mass index. *IQR* interquartile range. *ECOG* Eastern Cooperative Oncology Group. *CrCl* creatinine clearance

All patients received subcutaneous formulations of nadroparin as initial treatment. After 3 to 5 days, 99 patients transitioned to rivaroxaban as sequential therapy, and 118 patients continued with therapeutic dose nadroparin. All patients finished at least 90 days of therapeutic anticoagulation. A total of 183 (84.3%) patients received prolonged anticoagulation with a preventive dose, of whom 73 (33.6%) received 90 to 180 days, and the remaining 110 (50.7%) patients finished a total of 180 days anticoagulation therapy.

We also exhibited the demographics and comorbidities of the included patients according to different anticoagulants in Table 1. As our trial was not randomize-control designed, demographic differences were unsurprisingly present between patients treated with different sequential anticoagulants.

The catheters were kept for a median duration of 185 (IQR 132, 278) days before removal. Ten patients had their catheters removed within 90 days after the diagnosis of UEDVT; of them, 9 had suspected exit-site infections, 1 was in cancer remission, and the catheter was no longer needed. Although the catheters were removed, these 10 patients still finished their 90-day anticoagulation treatment.

### Recurrent VTE and bleeding event outcomes

Four (1.8%) patients with recurrent VTE were observed in 180 days. The cumulative rates of recurrence at 180 days were 1.7% and 2.0% in patients receiving nadroparin and rivaroxaban, respectively. This difference was not statistically significant (*p* = 0.777). Among the recurrent cases, 3 cases were symptomatic DVT located in distal lower limbs, and 1 case was located in the ipsilateral internal jugular vein. No PE event or catheter dysfunction was found and their catheter related DVT symptoms were resolved without local extension. The median time from initial anticoagulation to VTE recurrence was 45 days (range from 34 to 65 days). As the catheters were still functional and necessitated ongoing anticancer therapy, the catheters were kept in place, and all patients with recurrent VTE received unadjusted therapeutic dose of anticoagulants under close surveillance for a median of 147 days (range from 127 to 180 days). If any sign of further deterioration was found, the catheter would be removed and the anticoagulation would be adjusted. Deep vein recanalization was found in all 4 patients with monthly ultrasound monitoring, and no VTE progression was observed during the follow-up (Table 2).

**Table 2:** Details of recurrent VTE events

Case	anticoagulant	Recurrence location	Occurred at days of anticoagulation	Total anticoagulation duration(days)
1	nadroparin	Distal lower limbs	34	127
2	rivaroxaban	Distal lower limbs	39	157
3	rivaroxaban	Distal lower limbs	41	137
4	nadroparin	Ipsilateral IJV	65	180

*IJV* internal jugular vein

We also studied bleeding events during anticoagulation therapy. The overall bleeding rate at 180 days was 8.8%. No major bleeding event was observed in 180 days. CRNMB events occurred in 19 (8.8%) patients, presenting with hematuria (6 patients, 2.8%), gastrointestinal bleeding with positive fecal occult blood (5 patients, 2.3%), subcutaneous hemorrhage (4 patients, 1.8%), hemoptysis (2 patients, 0.9%) and epistaxis (2 patients, 0.9%) (Table 3). Ten patients who already received anticoagulation for at least 90 days stopped anticoagulation immediately after CRNMB and were followed with monthly serial imaging for PICC-related UEDVT. The remaining 9 patients decreased to a preventive dose anticoagulation until completion of 90 days therapy. No more bleeding events or VTE progression were observed during the follow-up.

**Table 3:** Details of bleeding events

Case	Anticoagulant	Occurred at days of anticoagulation	Tumor type	Bleeding site
1	Nadroparin	137	gastrointestinal	gastrointestinal
2	Nadroparin	64	lymphoma	subcutaneous
3	Rivaroxaban	139	lymphoma	subcutaneous
4	Nadroparin	106	breast	hematuria
5	Rivaroxaban	44	gynecology	hematuria
6	Rivaroxaban	65	gastrointestinal	gastrointestinal
7	Rivaroxaban	61	lung	epistaxis
8	Rivaroxaban	83	breast	hematuria
9	Rivaroxaban	98	breast	subcutaneous
10	Rivaroxaban	181	breast	hematuria
11	Rivaroxaban	63	breast	gastrointestinal
12	Nadroparin	163	breast	gastrointestinal
13	Nadroparin	152	breast	hematuria
14	Rivaroxaban	26	gynecology	gastrointestinal
15	Rivaroxaban	179	gastrointestinal	subcutaneous
16	Nadroparin	110	gynecology	hematuria
17	Rivaroxaban	39	gastrointestinal	epistaxis
18	Rivaroxaban	163	lung	hemoptysis
19	Rivaroxaban	79	lung	hemoptysis

**Fig. 2:**
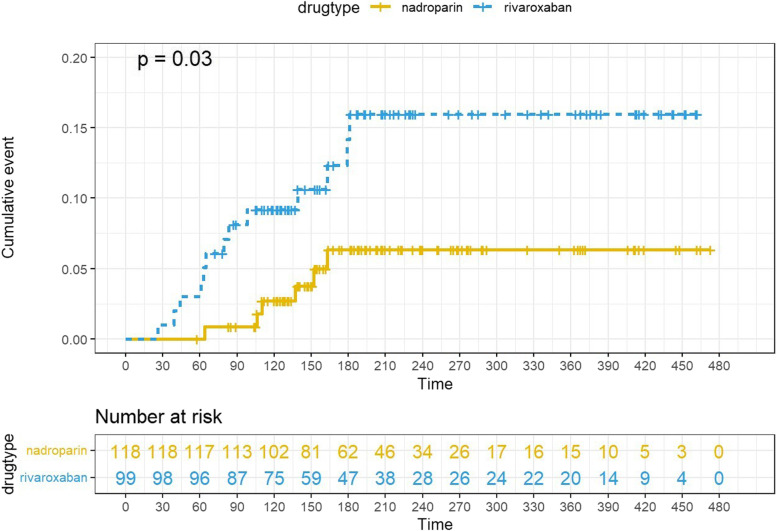
Cumulative bleeding events by univariate survival analysis

**Fig. 3:**
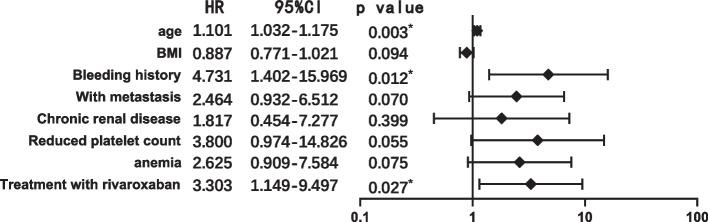
Multivariate Cox proportional hazard regression analysis of factors associated with bleeding events at 180 days. BMI, body mass index; CI, confidence interval; HR, hazard ratio; ^*^Statistical significance at a 95% confidence level

Patients in the rivaroxaban group had a higher 180-day cumulative bleeding event probability on Kaplan‒Meier analysis than those in the nadroparin group (13.1% for rivaroxaban, 5.1% for nadroparin, *p* = 0.03) (Fig. 2). Univariate analysis was performed and factors with statistically significant differences (*p* < 0.10) were included for multivariate analysis (Table 4). Further Cox proportional hazards analysis manifested that older age (hazard ratio [HR]: 1.101, 95% CI 1.032–1.175, *p* = 0.003), bleeding history (HR: 4.731, 95% CI 1.402–15.969, *p* = 0.012) and treatment with rivaroxaban (HR: 3.303, 95% CI 1.149–9.497,* p* = 0.027) were significant predictors of bleeding at the 180-day follow-up. The results of our proportional hazards model are listed in Fig. 3.

**Table 4:** Univariate analysis in patients with/without bleeding events

	**Non-bleeding group (** ***n*** ** = 198)**	**Bleeding group (** ***n*** ** = 19)**	***p*** ** value**
**Age, median(IQR)**	56 (47–62)	63 (53–65)	0.007
**Male gender (%)**	87 (43.9%)	5 (26.3%)	0.138
**BMI,median(IQR)**	23.1 (21.5–26.9)	21.2 (18.7–24.0)	0.032
**With other site VTE**			
** Pulmonary thromboembolism**	6 (3.0%)	2 (10.5%)	0.148
** Lower extremity Deep vein thrombosis**	14 (7.1%)	3 (15.8%)	0.176
** VTE history**	12 (6.1%)	1 (5.3%)	1.000
** Bleeding history**	7 (3.5%)	4 (21.1%)	0.009
**Primary cancer**			0.711
** Breast**	52 (26.3%)	7 (36.8%)	
** Lymphoma**	38 (19.2%)	2 (10.5%)	
** Gastrointestinal**	57 (28.8%)	4 (21.1%)	
** Gynecology**	22 (11.1%)	3 (15.8%)	
** Lung**	29 (14.6%)	3 (15.8%)	
** With metastasis (%)**	78 (39.4%)	12 (63.2%)	0.045
**ECOG performance**			0.627
** 0**	56 (28.3%)	4 (21.1%)	
** 1**	82 (41.4%)	10 (52.6%)	
** 2**	60 (30.3%)	5 (26.3%)	
**Antiangiogenic** **therapy used**	51 (25.8%)	2 (10.5%)	0.171
**Underlying diseases/conditions**			
** Cardiovascular disease**	37 (18.7%)	4 (21.1%)	0.763
** Diabetes**	19 (9.6%)	3 (15.8%)	0.419
** Abnormal Renal function (30 ml/min ≤ CrCl < 60 ml/min)**	9 (4.5%)	3 (15.8%)	0.076
** Reduced platelet (Platelet count < 100 × 10^9/L)**	10 (5.1%)	3 (15.8%)	0.093
** Anemia(Hemoglobin < 130 g/L for man, < 120 g/L for woman)**	29 (14.6%)	6 (31.6%)	0.055
** Non-single lumen PICC**	8 (4.0%)	0	1.000
** Left arm insertion**	31 (15.7%)	3 (15.8%)	1.000
** Exit site infection**	7 (3.5%)	2 (10.5%)	0.181
** Treatment pattern (LMWH)**	112 (56.6%)	6 (31.6%)	0.037
** Anticoagulation duration (days), median (IQR)**	180 (125–180)	163 (128–1)	0.725
**Outcome**			
** Recurrent VTE**	3 (1.5%)	1 (5.3%)	0.309
** Death from any cause**	3 (1.5%)	0	1.000

## Discussion

Patients with cancer are at increased risk of thrombosis, particularly those with a central venous device [10–13]. The incidence of UEDVT is increasing due to the widespread use of PICCs and improved survival of patients with cancer. Symptomatic catheter-associated thrombosis occurs in 3.0–5.0% of patients with cancer requiring venous access, which may increase to as much as 30.0% when including asymptomatic cases [12, 14].

However, the management of PICC-related UEDVT in cancer patients may be more complicated than in the general population given the higher risk of recurrent thrombosis and anticoagulation-associated bleeding experienced by cancer patients [15–18]. In the noncancer population, the ACCP 2016 guidelines recommended direct oral anticoagulants (DOACs) over vitamin K antagonist (VKA) therapy for VTE based on the greater convenience and abundant evidence that DOACs have similar efficacy in noncancer patients with fewer adverse events, such as life-threatening bleeding [19–22].

LMWH was recommended as the first-line choice of anticoagulant for cancer patients with VTE in major guidelines. From 2018 to 2020, several high-quality trials, including the Hokusai VTE, SELECT-D, ADAM VTE and Caravaggio studies, were published to compare DOACs with LMWH for the treatment of VTE in patients with cancer [2–5]. These head-to-head studies and subsequent meta-analyses indicated that DOACs were associated with lower VTE recurrence. In Caravaggio and ADAM studies, apixaban demonstrated similar or even lower incidence of bleeding events compared to LMWH. However, results from SELECT-D and Hokusai studies raised concern about the increased risk of bleeding in patients treated with rivaroxaban and edoxaban. Based on these results, contemporary guidelines, including the most recent NCCN guidelines, cite DOACs as an acceptable option for VTE treatment in cancer patients [6, 7, 19].

In contrast to classic LMWH and VKA, DOACs need no daily injection and routine monitoring, and the extensive experience in treating PE and lower extremity DVT using DOACs in cancer patients indicated that DOACs may be an alternative option for the long-term management of UEDVT in cancer patients. However, there are only sporadic reports about the possible use of DOACs in cancer patients with PICC-related UEDVT. Limited randomized controlled trials have focused on the management of this topic, and most recommendations are based upon observational studies or extrapolation from studies of noncatheter-related lower-extremity deep vein thrombosis (LEDVT). The optimal choice and duration of PICC-related UEDVT in cancer patients are still unclear.

In our study, the 180-day cumulative risk of recurrent VTE in the cancer patients receiving rivaroxaban was comparable with those receiving nadroparin (1.7% vs 2.0%, *p* = 0.777). Compared to the recurrence of 7–11.1% demonstrated in previous studies of LMWH treatment in cancer-associated thrombosis (CAT), the recurrent VTE rate at 180 days in our study was at a relatively low level of 1.8%, which was consistent with the results of studies focusing on UEDVT treatment in cancer patients. In the catheter 2 study that assessed rivaroxaban monotherapy in cancer patients with UEDVT, seventy cancer patients were included with a mean age of 54 years, and the most common malignancy was breast cancer (41%). All patients were treated with rivaroxaban for 12 weeks; the recurrent VTE at 12 weeks was 1.43% [23]. Similar low recurrence rate was also seen in the Catheter study and a recent meta-analysis [24]. In a meta-analysis published in 2021, a pooled analysis from 7 trials with 100% cancer patients and an indwelling catheter showed that the recurrent VTE rate was 1.7% (8/468) [1, 25].

UEDVT is usually excluded from large clinical trials of anticoagulants for VTE treatment. In comparison to usual site VTE, PICC-related UEDVT is often provoked by unique risk factors determining incidence and recurrence. The presence of an indwelling catheter in the upper arms represents a local and transient additional thrombotic risk factor in cancer patients because of vessel wall mechanical damage and blood coagulation activation from infused medications. This may partially explain why the outcome from our analysis was different from other data focusing on cancer patients treated for usual site VTE in the literature.

Another important objective of our study was to assess the rates of all bleeding events during anticoagulation.

In our study, although there was no major bleeding event observed at 180 days of anticoagulation, Kaplan‒Meier analysis demonstrated a significant difference in CRNMB rates at 180 days. Adjusted for baseline characteristics, the Cox proportional hazard model showed a higher CRNMB risk in the rivaroxaban group than in the nadroparin group (HR 3.30, 95% CI 1.15–9.50, *p* = 0.03).

In the two previous cited studies evaluating DOACs for PICC-related UEDVT in cancer patients, the bleeding results were similar to our study [23, 25]. In the first study comparing 44 patients treated with rivaroxaban to 40 patients treated with enoxaparin/warfarin, nonmajor bleeding events occurred in 7.3% and 11.4% of the rivaroxaban- and enoxaparin/warfarin-treated patients, respectively. In the Catheter 2 study, all bleeding events occurred in 13% (7 major bleeding and 4 CRNMB) [23].

Several limitations in our study should be acknowledged. First, given the inherent limitation of the retrospective and nonrandomized design of this study, loss of follow-up and selection bias could have been introduced into our cohort. Our study focused on PICC-related UEDVT in cancer patients, the included patients were homogeneous in terms of baseline characteristics, underlying UEDVT risk factors, anticoagulant treatment and duration of follow-up. As we mentioned before, the data of this cohort were derived from a prospective electronic registry database designed for central venous access administration, accompanied as an important part of anticancer therapy, and only 4.7% (11/233) of patients were lost to follow-up.

Second, our study had a relatively small sample size. Although catheter-related UEDVT accounts for 50–90% of all UEDVT cases, recent studies reported that the incidence of UEDVT was just 4–10% of all newly diagnosed VTE cases [16]. Available data on DOACs for UEDVT in cancer patients are still very scarce. In a recently published meta-analysis assessing anticoagulation for UEDVT, 1473 patients from 20 studies were included, and the average number of enrolled patients in each study was no more than 80 (range from 10 to 210) [1].

Third, we included only symptomatic UEDVT patients, which may have underestimated the incidence of all UEDVT cases. However, many impressive studies for VTE treatment (such as SELECT-D, Hokusai-VTE, Caravaggio studies) set symptomatic or incidental VTE as inclusion criteria, and routine ultrasound surveillance for DVT is also not recommended in major guidelines unless there are clinical signs for VTE [3–5]. In our Venous Assess Center, consultations for suspected UEDVT by specialist PICC nurses were provided when patients came to our center for weekly dressing changes. Further imaging examination was arranged once any clinical signs were present.

## Conclusion

Our study supported the efficacy of rivaroxaban for treating PICC-related UEDVT in cancer patients. However, compared with usual site VTE, data on anticoagulation therapy for PICC-related UEDVT in cancer patients are different, presenting with a low risk of VTE recurrence and a relatively high risk of clinically relevant nonmajor bleeding events. Considering the risk–benefit ratio, further well-designed trials are required to optimize the drug selection and duration for the treatment of PICC-related UEDVT in cancer patients.

## Data Availability

The datasets used in the current study are available from the corresponding author on reasonable request.
